# Drug-Coated Balloons in Percutaneous Coronary Intervention: Beyond Evidence

**DOI:** 10.7759/cureus.72990

**Published:** 2024-11-04

**Authors:** Nagi Azzi, Randa N Tabbah

**Affiliations:** 1 Cardiology, Lebanese American University School of Medicine, Beirut, LBN; 2 Cardiology, Notre Dame des Secours University Hospital, Byblos, LBN; 3 Cardiology, University of Balamand, Beirut, LBN

**Keywords:** drug-coated balloon (dcb), drug-eluting stent (des), leave nothing behind technique, paclitaxel-coated balloon, sirolimus-coated balloon

## Abstract

Drug-coated balloons (DCBs) represent a promising alternative to drug-eluting stents (DESs) by adopting a “leave-nothing-behind” approach, avoiding the long-term implantation of metallic materials associated with vessel damage, stent thrombosis, and restenosis. Although DCBs have historically been indicated for patients with in-stent restenosis or de novo small-vessel disease, recent studies indicate the potential for broader applications in acute coronary syndromes (ACSs). Trials comparing DCB and DES treatments show that DCBs yield comparable long-term outcomes, with some studies suggesting reduced rates of secondary endpoints such as cardiac death and non-fatal myocardial infarction among ACS patients treated with DCBs.

Research on DCBs in various clinical scenarios, including ST-segment elevation myocardial infarction and non-ST-segment elevation myocardial infarction, has shown promising results for safety and efficacy, with particular support for combining DCBs with other techniques such as excimer laser coronary atherectomy. These findings support the viability of DCBs as an effective alternative to DES for select patients. Technical guidelines and best practices for optimal DCB application have also emerged, emphasizing lesion preparation and drug delivery techniques. Further studies are anticipated to provide deeper insights into the long-term outcomes and broader clinical applications of DCBs, potentially establishing them as a primary approach in interventional cardiology.

## Introduction and background

The safety and effectiveness of percutaneous coronary intervention (PCI) for the treatment of obstructive coronary artery diseases (CADs) is confirmed by the sharp decline in the risk for both restenosis and stent thrombosis observed with drug-eluting stents (DESs). However, one intrinsic drawback of the metallic stent is that it involves foreign material within the native coronary artery, which can lead to neo-atherosclerosis, vascular inflammation, hypersensitivity, and stent thrombosis [1,2]. This permanent “iron cage” impairs the restoration of vasomotor function and the late luminal enlargement.

Therefore, a drug-coated balloon (DCB) is a desirable substitute for DES as it uses a lipophilic matrix to transport antiproliferative medications (such as sirolimus or paclitaxel) directly to the vascular wall, doing away with the necessity for a long-term carrier such as a robust polymer or metallic prosthesis. Although broader clinical indications are being explored, patients with in-stent restenosis (ISR) or de novo small-vessel disease (SVD) have been the focus of the research on this technology’s safety and effectiveness [1]. Current DCBs are semi-compliant and use an excipient to retain the drug. During inflation, a rapid delivery of the drug is noticed into the coronaries [2,3]. Moreover, DCBs employ a “leave-nothing-behind” technique. In contrast, with DES, a metallic material is left behind, contributing to vessel damage, stent thrombosis, and restenosis [3,4].

This study aims to determine whether DCB will replace DES in its main indications or does this hypothesis needs to be refined with further studies.

## Review

This review summarizes new data on DCBs from randomized trials and meta-analyses published between 2019 and 2024. The findings suggest a potentially transformative role for DCBs in interventional cardiology, with specific considerations and ongoing trials that will further clarify their use.

The population studied in this review is patients (both males and females) with CAD and ACS aged 50 years and more. This analysis aims to shed light on the importance of DCB and its specific indications.

Latest studies on DCB use in their known indications: SVD and ISR

In 2022, the BASKET-SMALL 2 study randomly assigned 758 patients with small CAD to two groups, namely, DCB or DES treatment. The patients were followed up for three years for major adverse cardiac events such as cardiac mortality, non-fatal myocardial infarction (MI), and target vessel revascularization [1]. No interaction was noticed between the indication for PCI (acute versus chronic coronary syndrome) and the therapeutic effect of DCB versus DES in patients with small-vessel CAD in a subgroup analysis [1]. For cardiac death (P for interaction, 0.049) and non-fatal MI (P for interaction, 0.010), a significant interaction between clinical presentation and treatment was seen at one year with lower rates of these secondary endpoints in patients with ACS treated by DCB.

A recent systematic review and meta-analysis by Murphy et al. conducted the long-term monitoring of DCBs and DESs in small coronary arteries (<3 mm), revealing that DCBs are equivalent to DESs in terms of all measured outcomes after one, two, and three years of follow-up. The DCB arm of the BASKET-SMALL 2 study showed a noteworthy decrease in the incidence of non-fatal MI after one year. Additionally, there was a reduction in severe bleeding episodes after two years. The study illustrated the potential long-term usefulness of novel DCBs in small CAD revascularization [5].

Concerning ISR, a recent meta-analysis of 16 randomized controlled trials (RCTs) comparing DCBs and DESs in managing ISR patients found that DCBs significantly reduced the risk of major adverse cardiac events (MACE) and late lumen loss (LLL) compared to DESs. The study included 2,686 patients and found that DCBs significantly decreased the risk of MACE and LLL compared to DESs. The risk of in-stent binary restenosis was also lower in the DCB intervention compared to DES. This meta-analysis further confirmed DCBs as an alternative approach to DESs in the management of ISR [6].

Latest studies on DCB in ACS

The REVELATION (REVascularization With PaclitaxEL-Coated Balloon Angioplasty Versus Drug-Eluting Stenting in Acute Myocardial Infarction) trial found that, in the setting of ST-elevation myocardial infarction (STEMI), a DCB strategy was non-inferior to a DES strategy in terms of fractional flow reserve (FFR) at nine months. The two-year clinical outcome of this study was excellent and comparable between the DCB and groups [7].

A recent study by Tonomura et al. enrolled 62 patients with STEMI within 24 hours of symptom onset. The study examined the feasibility of combining excimer laser coronary atherectomy (ELCA) and DCB angioplasty for clinical outcomes. The study found no adverse events, and minimal lumen diameters were almost identical after the procedure. target lesion revascularization (TLR) was performed in 8.1% of patients. Dual antiplatelet therapy was administered for 2.3 ± 2.2 months, and no abrupt vessel closure, reinfarction, cardiac death, or major bleeding was observed [8].

Merinopoulos et al. recently evaluated the safety of DCB-only angioplasty against second-generation DES in primary PCI. For a median follow-up duration of >3 years, 452 patients with STEMI due to de novo illness were treated with DCBs and 687 with DESs. There was no difference in mortality between DCBs and DESs. The study indicated that DCB-only angioplasty appeared safe in all-cause mortality and net adverse cardiac events, including unplanned TLR compared to DES for STEMI. In certain patient populations, DCB may be an effective and safe alternative to DES [9].

The long-term safety and efficacy of the paclitaxel-DCB after a bare-metal stent (BMS) implantation for STEMI at baseline (DCB-combined strategy) is uncertain. The PEBSI-1 trial, involving patients with STEMI, aimed to evaluate the effectiveness of a DCB-combined or BMS-only strategy [10]. The primary endpoint at the ninth-month follow-up was in-stent LLL [10]. At eight years, the DCB-combined strategy group had a lower target vessel revascularization (TVR) rate and a trend toward lower TLR. However, the BMS group experienced additional cardiovascular death, TVR, TLR, and stent thrombosis. The DCB-combined strategy for STEMI patients supports the safety and efficacy of this technique. The lowest rates of TVR, TLR, and very late stent thrombosis (VLST) were found in this trial. More studies are needed to evaluate its superiority compared to DES, specifically the new generations, and the likelihood of preventing very late events in this category of patients [10].

Furthermore, Wang et al. included 1,032 instances of type 1 MI and 42 cases of type 4C MI, a subtype of MI associated with restenosis without thrombosis. MACEs were more common in people with type 4C MI. According to the study, type 4C MI had a worse prognosis than type 1 MI. In the treatment of type 4C MI, DCB angioplasty and DES implantation had comparable effectiveness [11] (Table 1).

**Table 1:** Studies on DCB with related indications. ACS: acute coronary syndrome; BMS: bare metal stent; CAD: coronary artery disease; DCB: drug-coated balloon; DES: drug-eluting stent; ELCA: excimer laser coronary atherectomy; FFR: fractional flow reserve; ISR: in-stent restenosis; MACE: major adverse cardiac event; MI: myocardial infarction; NSTEMI: non-ST-elevation myocardial infarction; PPCI: primary percutaneous coronary intervention; STEMI: ST-elevation myocardial infarction

Study	Indication	Conclusions
BASKET-SMALL 2 Trial	DCB vs. DES in small-vessel disease	Lower rates of secondary endpoints were seen at one year in patients with ACS treated with DCB
Murphy et al. (meta-analysis)	DCB vs. DES in small CAD <3 mm	DCB and DES were equal in terms of all measured outcomes after one, two, and three years with a decrease in non-fatal MI at one year
Aziz et al. (meta-analysis)	DCB vs. DES in ISR	DCB decreased MACE and lowered ISR
REVELATION trial	DCB (paclitaxel) vs. DES in STEMI	DCB was non-inferior to DES in terms of FFR and outcomes were excellent at two years
Tonomura et al.	DCB with ELCA in STEMI	No major events were noticed
Merinopoulos et al.	DCB (paclitaxel) only in STEMI and PPCI vs. DES	No difference in safety compared to DES for STEMI and a good alternative to DES in select populations
PEBSI study	DCB-combined strategy (paclitaxel) vs. BMS-only for STEMI	Safety and clinical efficacy over time for the combined strategy (long-term efficacy)
Wang et al.	DCB vs. DES in type 4C MI	DCB angioplasty and DES were comparable

Finally, a recent meta-analysis of 13 studies comparing DCBs and DESs in patients with acute MI undergoing PCI found that DCBs were not inferior to DESs in terms of MACE, cardiac death, all-cause death, myocardial infarction, TLR, stent thrombosis, LLL, and minimum lumen diameter (MLD) [6]. This analysis revealed a non-inferiority of DCB over DES in terms of MACE. Moreover, DCB was not inferior to DES in terms of all-cause mortality, cardiac mortality, MI, stent thrombosis, TLR, LLL, or MLD [6]. The meta-analysis concluded that DCB was not inferior to DES in patients with acute MI and could be recommended as a likely strategy in this population [6].

Technical considerations

A clinical expert consensus document on DCB for CAD from the Japanese Association of Cardiovascular Intervention and Therapeutics proposed a step-by-step approach for the DCB-only strategy. It concluded that the use of DCB is increasing in clinical settings, facilitating the “leave-nothing-behind” strategy. However, clinical indications beyond classical ISR or SVD need further evaluation, and additional scientific evidence is needed for de novo CAD treatment [12,13].

A step-by-step approach for the DCB-only strategy was suggested. The first step is to evaluate the suitability for DCB; these patients need to have multiple stents. Concerns about the presence of a metallic material for a long time in patients with allergies to possible materials and the young population should be considered. Moreover, the bleeding risk should be taken into consideration for those who are at high risk.

Regarding the angiographic conditions, several issues were also considered. The best category would be those with ISR and SVD, as indicated in several trials. In addition, ostial lesions, bifurcations with a need for side branch dilatation, and nodular calcified lesions are not anticipated to be well expandable.

Going deeper into the interventional techniques, a second step is the optimal preparation of the lesion before using a DCB. First, a pre-dilatation with a cutting or scoring balloon is advised with a 1:1 balloon-to-artery ratio. Intracoronary imaging is always encouraged. If moderate-to-severe calcification is present, then adjunctive atherectomy or lithotripsy should be considered.

The third step is the assessment after pre-dilatation with a Thrombolysis in Myocardial Infarction grade 3 flow (non-flow limiting), angiographic residual stenosis of 30%, and, especially, the absence of major dissection and findings suggestive of intraluminal thrombus is recommended. If all these criteria are satisfied, the DCB-only approach is warranted. If not, then a bailout strategy by DES is indicated.

Of note, these balloons have some specific considerations. To ensure an acceptable drug deployment and good adherence to the wall it is mandatory to maintain the DCB inflated for at least 30 seconds to warrant adequate drug transfer to the lesion. Furthermore, inflating the balloon with higher pressure could cause a coronary dissection leading to a stent bailout implantation [14]. Finally, this approach seems attractive and practical, especially as it derives from an internationally recognized clinical expert consensus.

Beyond evidence: paclitaxel versus sirolimus DCB

The TRANSFORM I trial studied the sirolimus-coated balloon( SCB) versus the paclitaxel-coated balloon (PCB) in de novo small vessels. Coronary angioplasty outcomes at six months were assessed after the treatment of de novo small vessels with SCB or PCB. This prospective study of 121 patients is a non-inferiority trial with balloon sizing using optical coherence tomography (OCT) and net lumen gain at six months. The Magic Touch SCB failed to demonstrate the non-inferiority to the Sequent Please Neo PCB for angiographic net lumen gain at six months [15].

The AGENT IDE trial studied 600 patients with a mean age of 68 years and followed them for 12 months. The main purpose of this study was to assess the efficacy and safety of the PCBs in subjects with ISR. The study illustrated that DCB is superior to balloon angioplasty in decreasing treatment failure in patients with coronary ISR (p = 0.003) [16]. There was a decrease in repeat revascularization (p = 0.001), target vessel MI (p = 0.02), and stent thrombosis. AGENT DCB had a lower paclitaxel dosage than the Sequent DCB (2 µg/m^2^ vs 3 µg/m2) One important limitation of this study was that they did not study the balloon versus DES. The FDA approved AGENT PCB to treat ISR in March 2024.

However, there were some inclusion criteria of this study we need to be aware of and follow closely. Patients had ISR in lesions with a previous BMS or DES with lesion length <26 mm, vessel diameter >2.0 to ≤4.0 mm, and degree of stenosis >70 to <100% in asymptomatic patients or >50 to <100% in symptomatic population [16].

As mentioned before, the AGENT IDE did not compare DCB with DES. Hence, further studies are needed for this. TRANSFORM II is an ongoing study emphasizing this comparison to confirm the hypothesis suggesting a non-inferior to a current DES for patients with de novo lesions in coronary vessels with a diameter of 2-3 mm [17].

Cost-effectiveness analysis

Merinopoulos et al. performed a cost analysis study of DCB-only angioplasty versus DES for de novo disease of all vessel sizes and all clinical indications. The study compared the total costs of patients treated with DCB or DES for first-presentation STEMI, NSTEMI, or stable angina due to de novo disease. Results showed that DCB patients were £9.02 more expensive per patient, but cheaper per lesion treated. The cost minimization analysis showed consistent results regardless of long-term antiplatelet medication duration. Finally, this cost-effectiveness comparison showed that the per-patient and per-lesion results were not different; hence, cost should not be implicated in the decision to choose DCB or DES [18].

Drawbacks or limitations

Drawbacks were seldom noted, for example, in a meta-analysis of three RCTs done by Changal et al. The trial aimed to illustrate the efficacy of DCBs versus DES in STEMI. No significant difference was seen between DCB and DES for MI (1.4% vs. 1.3%, p = 0.98). More coronary dissections were noticed with DCBs (p = 0.0006), and bailout stenting with a BMS was required in 18 (13.0%) patients. No sufficient evidence was seen with DCB in STEMI patients, and further data are needed to confirm this [3].

Future directions

PCBs are only recommended in selected indications. The SELUTION DeNovo trial (sirolimus-eluting balloon treatment) is a multi-centric international randomized trial comparing a technique of PCI with SELUTION SLR TM 014 PTCA balloon (SEB) and provisional DES to a strategy of PCI with systematic DES on target vessel failure. The trial aims to determine if the SELUTION DeNovo strategy is non-inferior to the systematic DES strategy on TVF at those milestones. The trial will include 3,326 patients in Europe and Asia, with TVF rates and components determined up to five years post-intervention [19].

Finally, a PCR statement from the European Society of Cardiology on interventions with DCBs was published on May 17, 2023. It noted that the community is increasingly interested in using DCBs for treating de novo disease, particularly in lesions with diffuse disease in diabetes mellitus, multivessel disease, ACS, and high bleeding risk patients [20]. The idea of “leaving nothing behind” and the possibility of long-term preservation of the physiological vessel components are appealing. Therefore, the “hybrid approach” that combines DCB and DES seems fascinating.

Clinical evidence for DCB in de novo disease related to small vessel disease is scarce, with modest-sized trials and variable quality metrics. The international communities are encouraging to enhance the evidence base for DCB, especially in patients with de novo disease. A huge effort is needed to coordinate resources, identify unmet needs, and explore the best funding model for trials in the future [20].

## Conclusions

The DCB is the way forward with its well-defined indications and technical approach described in this article. The previous experience with bioresorbable scaffolds was deceiving, while DCBs were not adequately explored. The idea from the start was to mimic the DES concept by replacing it with a scaffold that could “disappear” afterward from the arteries. However, that did not happen as expected because it finally disappeared from hospital shelves. Let’s hope this time, as we are confident, we are on the right track to create a more comprehensive approach to CAD. A PCB is now FDA-approved for ISR. Further studies are needed to ensure the safety and efficacy of DCBs in clinical practice.
